# Role of the Bacterial Amyloid-like Hfq in Fluoroquinolone Fluxes

**DOI:** 10.3390/microorganisms12010053

**Published:** 2023-12-28

**Authors:** Florian Turbant, Emeline Esnouf, Francois Rosaz, Frank Wien, Grzegorz Węgrzyn, Hugo Chauvet, Véronique Arluison

**Affiliations:** 1 Synchrotron SOLEIL, L’Orme des Merisiers, Saint Aubin BP48, 91192 Gif-sur-Yvette, France; florian.turbant@synchrotron-soleil.fr (F.T.); frank.wien@synchrotron-soleil.fr (F.W.); hugo.chauvet@synchrotron-soleil.fr (H.C.); 2 Laboratoire Léon Brillouin LLB, UMR12 CEA CNRS, CEA Saclay, 91191 Gif-sur-Yvette, France; emeline.esnouf@supagro.fr (E.E.); francois.rosaz@cea.fr (F.R.); 3 Department of Molecular Biology, Faculty of Biology, University of Gdansk, Wita Stwosza 59, 80-308 Gdansk, Poland; grzegorz.wegrzyn@ug.edu.pl; 4 UFR SDV, Université Paris Cité, 75013 Paris, France

**Keywords:** AcrAB-TolC drug efflux complex, ciprofloxacin, fluoroquinolone, Hfq protein, small noncoding RNA, outer membrane porin, single cell, antibiotic import and export, riboregulation

## Abstract

Due to their two-cell membranes, Gram-negative bacteria are particularly resistant to antibiotics. Recent investigations aimed at exploring new target proteins involved in Gram-negative bacteria adaptation helped to identify environmental changes encountered during infection. One of the most promising approaches in finding novel targets for antibacterial drugs consists of blocking noncoding RNA-based regulation using the protein cofactor, Hfq. Although Hfq is important in many bacterial pathogens, its involvement in antibiotics response is still unclear. Indeed, Hfq may mediate drug resistance by regulating the major efflux system in *Escherichia coli*, but it could also play a role in the influx of antibiotics. Here, using an imaging approach, we addressed this problem quantitatively at the single-cell level. More precisely, we analyzed how Hfq affects the dynamic influx and efflux of ciprofloxacin, an antibiotic from the group of fluoroquinolones that is used to treat bacterial infections. Our results indicated that the absence of either whole Hfq or its C-terminal domain resulted in a more effective accumulation of ciprofloxacin, irrespective of the presence of the functional AcrAB-TolC efflux pump. However, overproduction of the MicF small regulatory RNA, which reduces the efficiency of expression of the *ompF* gene (coding for a porin involved in antibiotics influx) in a Hfq-dependent manner, resulted in impaired accumulation of ciprofloxacin. These results led us to propose potential mechanisms of action of Hfq in the regulation of fluoroquinolone fluxes across the *E. coli* envelope.

## 1. Introduction

Antibiotic crisis is defined as the appearance of bacterial strains resistant to most, if not all, known antimicrobial compounds, thus making infections caused by microorganisms lethal for hosts [1]. After the era of the extensive use of antibiotics, it is now estimated that in the United States, 2.8 million infections with bacteria resistant to antibiotics occur each year, causing about 35,000 human deaths. Furthermore, it is assessed that the number of multi-drug resistance (MDR)-caused deaths will increase to about 700,000 worldwide annually by 2050 if no novel therapies are developed [1]. New ways to fight bacterial infections are thus of utmost importance.

Currently, about 15 families of antibiotics are used. Their actions fall within three main mechanisms involving the inhibition of peptidoglycan synthesis (β-lactams), protein synthesis (aminoglycosides, macrolides or tetracyclines), and DNA metabolism and repair (quinolones) [2]. The mechanisms of bacterial resistance are diverse and include mutations causing specific changes in antibiotic targets, production of enzymes that destroy antibiotics, or export of antibiotics across cell membranes [3]. For instance, fluoroquinolones (FQ) are broad-spectrum antibiotics that are used for the treatment of severe infections, and resistance to FQ is typically due to mutations in genes encoding the target enzymes, DNA gyrase and topoisomerase IV, or genes involved in efficiency of the drug fluxes [4].

The bacterial cell membrane is indeed the first layer of defense against antimicrobial agents. Because Gram-negative bacteria contain two cellular membranes, i.e., an inner membrane (IM) and outer membrane (OM), their resistance to antibiotics is generally more pronounced and difficult to combat relative to Gram-positive species [5]. Thus, regulation of OM components strongly influences the antibiotic resistance of Gram-negative bacteria.

Bacteria can modulate their gene expression profile and adapt their metabolism to resist toxic agents [6,7]. This regulation operates at two levels, either transcriptional or post-transcriptional [5,7]. The former allows for the precise control of gene activity with minimal loss of cellular energy, while the latter makes possible a very quick response to rapidly changing environmental conditions. Targeting post-transcriptional regulation thus provides one of the most promising approaches to fight resistance to antibacterial agents. This approach may consist of blocking small noncoding RNA (sRNA)-based regulation [8]. Many sRNAs regulate mRNA translation via base-pairing in Gram-negative bacteria [9]. Nevertheless, because sRNA:mRNA annealing is imperfect, the Hfq protein is needed for this regulatory process [10]. Specifically, Hfq is required as a cofactor to facilitate sRNA:mRNA annealing [11]. sRNA usually negatively regulates mRNA expression by sequestering the ribosome-binding site (rbs) and/or the AUG start codon, thus blocking mRNA translation and subsequently accelerating the degradation of naked (i.e., ribosome-free) mRNA [12,13]. Translation enhancement may also occur but it is less frequent [14]. Due to the diversity of their targets, sRNAs and Hfq are involved in numerous bacterial pathways, including virulence and pathogenicity [15,16]. For this reason, Hfq has attracted considerable attention [17,18,19].

Structurally, Hfq consists of two regions. The N-terminal region (NTR, ~65 amino acid residues) forms a toroidal-shaped homohexamer that has at least two RNA-binding sites on each face of the torus [20,21,22]. These two binding sites tend to be specific for A- or U-rich RNA [20,22,23]. Additionally, Hfq possesses an amyloid C-terminal region (referred to as CTR, ~38 amino acid residues) [24]. The role of this C-terminal region remains poorly understood. While some studies indicated that Hfq-CTR is dispensable for riboregulation, other evidence indicated that it could play a role in sRNA:mRNA annealing [25,26,27]. Furthermore, it could play a role in DNA metabolism [28].

The efficiency of antibiotic efflux that decreases antibiotic intracellular concentration is an important parameter in one mechanism of antibiotic resistance. This mechanism results from the activity of transmembrane efflux pumps that recognize and expel a broad spectrum of antibiotics [29,30]. The Resistance Nodulation Division (RND) efflux pump family is the best characterized among them [31]. In particular, the major *E. coli* RND efflux pump AcrAB-TolC plays a critical role in the emergence of multidrug-resistant phenotypes [32]. This pump is composed of three proteins: AcrB is an active drug transporter that functions with the proton-motive force across the IM; TolC forms an OM channel; and AcrA is a periplasmic linker [33,34,35]. Two studies have shown that Hfq plays a role in regulating *acrB* expression, and therefore, Hfq may contribute to antibiotic resistance [36,37]. Furthermore, Hfq modulates antibiotic import. Indeed, the expression of mRNAs encoding various outer membrane proteins is controlled by sRNA and Hfq. These include several genes coding for porins [38,39]. Nevertheless, the relative impact of Hfq on influx and efflux processes remains unknown. Here, we addressed this question using a quantitative analysis of fluoroquinolone accumulation both at the single cell and whole population level [40,41]. Fluoroquinolones (FQ) represent a set of the most-used antibiotics to treat infections due to their broad-spectrum activity, tissue penetration, and low toxicity [42] Different *E. coli* variants were analyzed to decipher the role of Hfq on FQ (represented here by ciprofloxacin, CIP) influx and efflux.

## 2. Materials and Methods

### 2.1. Chemicals

All chemicals, except otherwise stated, were purchased from Sigma-Aldrich (Saint Louis, MO, USA).

### 2.2. Bacterial Strains

All strains used in this study (Table 1) are derivatives of *E. coli* AG100 (*argE3 thi-3 rpsL xyl mtl supE44*) [43]. The reference strain AG100 is referred to as a wild-type (WT) throughout this manuscript. The AG100A strain devoid of the AcrB efflux pump (AG100 *acrB::kan*) was described previously [44]. The strains mutated in the *hfq* gene are AG100 *hfq::cm* (devoid of the functional Hfq protein) and AG100-∆*ctr*, producing a truncated form of the protein with only the first 72 amino acid residues that correspond to Hfq-NTR [28]. These strains were constructed by P1 transduction of *hfq* alleles (*hfq::cm* or ∆*ctr* = *hfq72-cm*) into the AG100/AG100A background. The *hfq* gene regions of the strains were sequenced after transduction to confirm the presence of the *hfq* alleles (*hfq*+, *hfq::cm* or ∆*ctr)*. For simplicity, *hfq::cm* and *hfq72-cm* are referred to as ∆*hfq* and ∆*ctr* throughout the manuscript. To reduce the expression of the *ompF* gene, encoding the OmpF porin, hfq^+^ bacteria were transformed with the pBRpLac-MicF plasmid (Amp^r^), bearing the gene for expression of the MicF sRNA [45]. Expression of *micF* was induced by adding 1 mM isopropyl-β-d-thiogalactopyranoside (IPTG) at 0.4 OD_600_ and cultivation until OD_600_ 0.6. The effect of MicF overproduction using this plasmid was quantified previously using a reporter strain bearing an *ompF*-*mCherry* fusion and MicF overproduction drastically reduced *ompF* expression compared with the pBRpLac empty plasmid [46]. Here, we confirm by dot-blotting using an anti-OmpF antibody (from rabbit) that the amount of OmpF is reduced by ~70–80% in our conditions of growth. Note that the regulation of *ompF* expression by MicF requires the presence of Hfq [47].

### 2.3. Preparation of the Bacterial Suspension

Strains (WT and mutants) were grown at 37 °C in LB medium supplemented with chloramphenicol (Cm), ampicillin (Ap), and/or kanamycin (Kan) when necessary. Overnight cultures were diluted 1:100 in fresh LB medium and bacteria were grown until OD_600_ 0.6. The cells were then collected by centrifugation at 6000× *g* for 10 min at room temperature.

### 2.4. Analysis of Ciprofloxacin Accumulation in Bulk by Spectrofluorimetry

Cells from cultures at OD_600_ 0.6 were concentrated 10-fold in NaPi-Mg buffer (50 mM sodium phosphate, 0.5 mM MgCl_2_, pH 7.0). Bacteria were then incubated for 5 and 15 min in the presence of ciprofloxacin (CIP) at 5 µM. The Ciprofloxacin concentration was chosen considering AG100 and AG100A strains’ minimum inhibitory concentration (MICs), reported previously [41,48]. Controls consisted of bacteria incubated without the antibiotic. Then, bacterial suspensions (800 µL) were centrifuged at 6000× *g* for 10 min at 4 °C to eliminate CIP. Pellets were lysed overnight at 4 °C in 500 μL of 0.1 M glycine-HCl at pH 3. The supernatants were collected and natural fluorescence of CIP was measured in bulk using an excitation λ_exc_ = 275 nm and an emission spectrum λ_em_ from 320 to 500 nm [40]. All measurements were made simultaneously in a 96-well plate using a fluorescence plate reader (Tecan Life Science, Männedorf, Switzerland). Results were normalized to the tryptophan signal and CIP concentrations in lysates and calculated according to the calibration curves (see Vergalli et al. for details [40]). As the tryptophan signal may slightly change in the ∆*hfq* strain [49], fluorescent units were normalized to 1 using the WT AG100 strain after 5 min of incubation with CIP as a reference. All data are presented as the average of at least quadruplicate analyses made with independent cultures. Results were obtained from *n* = 4 to 9 independent cultures. The *t*-test was used to assess significative differences between CIP concentrations to compare the strains. The *t*-test can be applied to a small sample size as long as the population is normally distributed [50], which is the case here (see Supplementary Figure S1).

### 2.5. Single Cell Micro-Spectrofluorimetry

Bacterial cells for spectrofluorimetry were prepared in the same way as for bulk fluorimetry, except that cells were resuspended in NaPi-Mg buffer at an OD_600_ of 4.8 (120 µL) and distributed on a 1 M sucrose cushion (165 μL). The suspensions were centrifuged at 9000× *g* for 10 min at 4 °C. The pellets were stored on ice up to 2 h. The pellets were then resuspended in 40 µL of NaPi-Mg buffer just before imaging, in the presence or absence of CIP. Next, 0.5 µL of resuspended pellets were squeezed between two quartz coverslips and analyzed using a DUV fluorescence automatized inverted microscope called TELEMOS on the DISCO beamline at the SOLEIL synchrotron (proposal 20210406). For quantification, a series of acquisitions using an excitation wavelength of λ_exc_ = 275 nm were taken. Two sets of bandpass filters were used, one with λ_em_ ranging from 329 to 351 nm for tryptophan and the other ranging from 420 to 480 nm for the CIP drug fluorescence [40]. The images were analyzed with Image J and with a Python script developed on the DISCO beamline (https://gitlab.synchrotron-soleil.fr/disco-beamline/bacteria-drug-uv-analysis, accessed on 25 December 2023) [51]. For each condition, two different localizations with approximately 20–30 bacterial cells per field of view were recorded and averaged. Furthermore, at least three independent cultures were analyzed for each strain. The one-way ANOVA analysis of variance followed by a Tukey’s post hoc test was used to compare the means between the groups at the last measurement time.

### 2.6. Analysis of Bacterial Survival by Fluorescence Imaging

To evaluate bacterial survival, the standard staining microscopy method using the kit LIVE/DEAD^TM^ BacLight^TM^ (Thermofisher, Waltham, MA, USA) was used. This staining test allowed the direct differentiation between the total and dead bacteria using two fluorescent probes: Syto 9 which stains total bacteria as it penetrates both membranes of living and dead cells, and propidium iodide which is selective to the damaged membrane of dead cells. Staining was performed according to the manufacturer’s protocol. The quantitative assessment of cell viability based on live/dead staining has been proven using a comparison with flow cytometry [52]. This lower viability was confirmed by plating both strains after 7 min of CIP exposure.

## 3. Results

### 3.1. The Absence of Hfq Increases Antibiotic Accumulation Independently of AcrB

As Hfq could simultaneously regulate both FQ influx and efflux, we first focused our analysis on CIP influx using a strain devoid of the AcrB efflux pump. The AcrAB-TolC drug-efflux complex consists of AcrA, AcrB, and TolC polypeptides that work together. If only one of these subunits is absent or damaged (here AcrB) the activity of the whole pump is affected, limiting the efflux of FQs and increasing accumulation.

**Figure 1:**
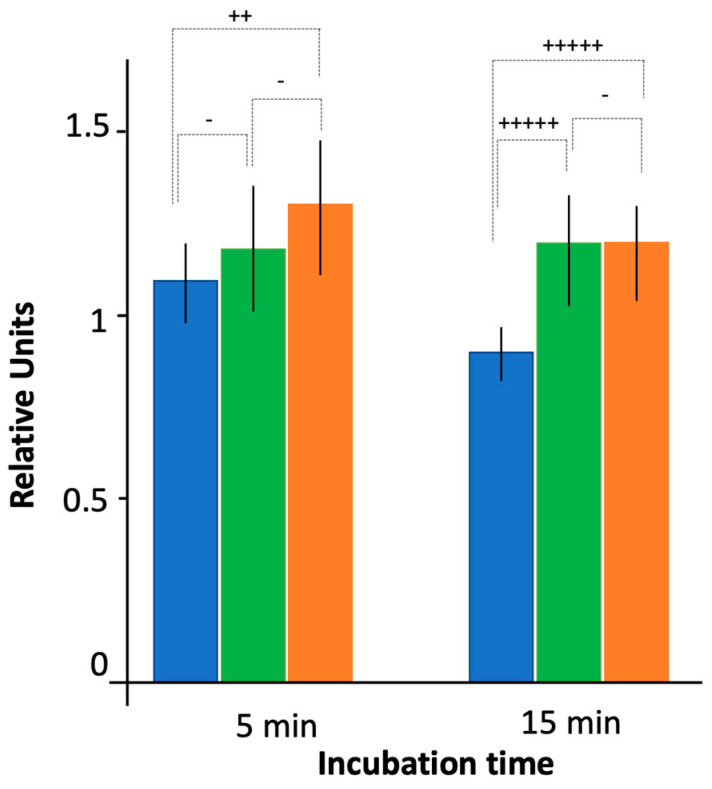
Ciprofloxacin accumulation by bacteria devoid of the efflux pump as assessed by fluorescence bulk measurements. Blue: AG100A *hfq*^+^; green: AG100A ∆*ctr*; orange: AG100A ∆*hfq*. Note that to compare results between the different conditions and experiments, fluorescent units were normalized to 1 using WT AG100 strain after 5 min of incubation with CIP as a reference (presented on Figure 4). Quantitatively, an exposure time to ciprofloxacin of 5 min for AG100 (our reference) corresponds approximately to 60,000 molecules/bacteria. The *t*-test was used to determine if the differences between intracellular CIP concentration for WT and mutated Hfq strains were significant (- *p* < 0.25; ++ *p* < 0.1; +++++ *p* < 0.01).

As shown in Figure 1, at the population level CIP accumulated more efficiently in the strain devoid of both Hfq and AcrB (AG100A ∆hfq) than in the strain only devoid of AcrB (AG100A). This is observed at 5 and 15 min. The accumulation ratio (e.g., the ratio of ciprofloxacin in AG100A hfq^+^/AG100A ∆hfq) was ~1.3 at 5 and 15 min. This effect was also observed for the ∆ctr strain, where Hfq devoid of its CTR-amyloid region is present, but the difference was less pronounced (accumulation ratio ~1.2), and the significance of the difference observed between ∆hfq and ∆ctr in the ∆acrB genetic background was not clear (*p* < 0.25). To complement and confirm measurements at the population level, we performed measurements at the single-cell level.

**Figure 2:**
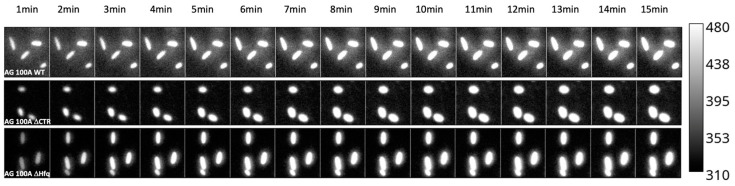
Time course accumulation of Ciprofloxacin at the single-cell level in bacteria devoid of the efflux pump. Kinetics were followed for 15 min at 20 °C using the bandpass filter 420–480 nm. As seen, bacteria retained their normal shape during the experiment.

The effects observed in bulk were confirmed by an analysis at the single-cell level (Figure 2). Compared with bulk measurements, this methodology offers the opportunity (i) to examine the time course accumulation without the need for bacterial synchronization; (ii) to follow the kinetics of accumulation in exactly the same bacteria through time; (iii) to observe the variability of accumulation within the same strain population; (iv) to monitor bacteria morphology during antibiotic accumulation.

**Figure 3:**
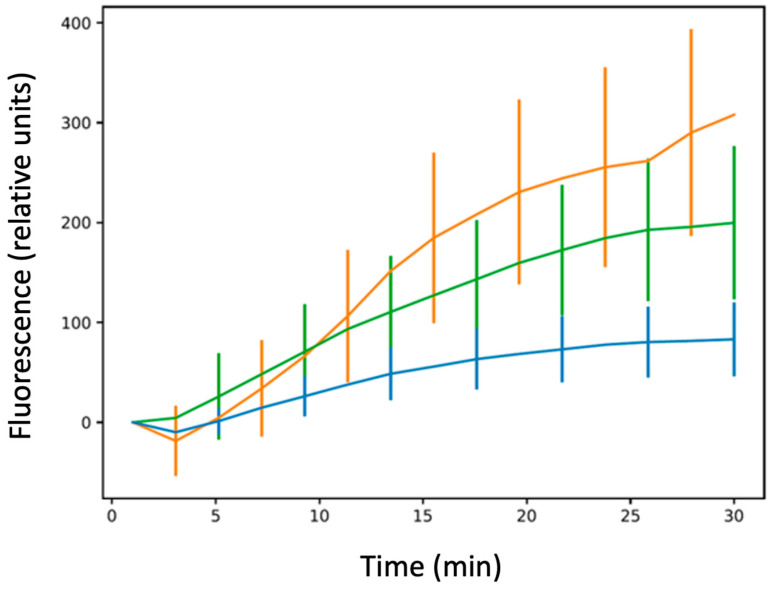
Ciprofloxacin accumulation at the single-cell level in bacteria devoid of the efflux pump. Blue: AG100A; green: AG100A∆*ctr*; orange: AG100A∆*hfq*. The results are shown as mean values from 20–30 bacteria analysed for at least three different pre-cultures, with error bars indicating SD. The one-way ANOVA analysis of variance followed by a Tukey’s post hoc test was used to compare the means between strains AG100A vs. AG100A∆*hfq*, AG100A vs. AG100A∆*ctr*, and AG100A∆*hfq* vs. AG100A∆*ctr* at the last recorded time point, which showed that the means of all pairs are significantly different with a *p*-value lower than 0.01. See also Supplementary Table S1.

As shown in Figure 2 and Figure 3, we confirmed that in the absence of Hfq, more CIP accumulated in bacterial cells relative to the *hfq*+ strain, similar to bulk measurements. We also confirmed that this effect was observed, even if less pronounced, for the ∆*ctr* compared with the ∆*hfq* strain, as suspected in bulk analysis.

### 3.2. The Absence of Hfq Increases Antibiotic Accumulation Also in the Presence of AcrB

**Figure 4:**
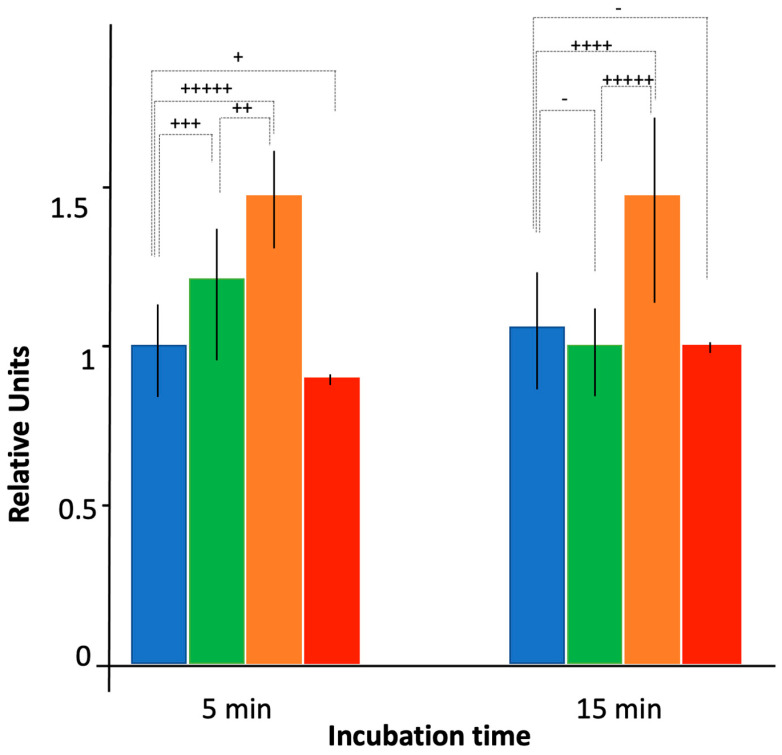
Ciprofloxacin accumulation as assessed by fluorescence bulk measurements. Blue: AG100; green: AG100 ∆ctr; orange: AG100 ∆hfq; red: AG100/pBRpLacMicF. As in Figure 1, fluorescence units were normalized to 1 using WT AG100 strain after 5 min of incubation with CIP as a reference. Quantitatively, the exposure time to ciprofloxacin of 5 min for AG100 corresponds approximately to 60,000 molecules per bacteria (our reference here in blue). The *t*-test has been used to determine if the differences between intracellular CIP concentration for WT and mutated Hfq strains are significant (- *p* < 0.25; + *p* < 0.2; ++ *p* < 0.1; +++ *p* < 0.05; ++++ *p* < 0.025; +++++ *p* < 0.01). Note that variances have similar values, except for the strain expressing MicF where the variance is very small, suggesting a part of the variability between cells comes from influx of CIP.

Using a bulk analysis (Figure 4), the absence of Hfq resulted in more effective CIP accumulation relative to the WT strain, bearing the functional efflux pump. The effect was similar to that observed for bacteria devoid of the efflux pump AG100A (see Section 3.1 and Figure 1). We observed for AG100A an accumulation ratio ~1.3, and this effect was also less pronounced with the ∆ctr strain than with full hfq deletion (accumulation ratio ~1.2 vs. 1.3, respectively). Here, a significant difference was observed between ∆hfq and ∆ctr mutants in the WT genetic background, in particular at 15 min (*p* < 0.01).

**Figure 5:**
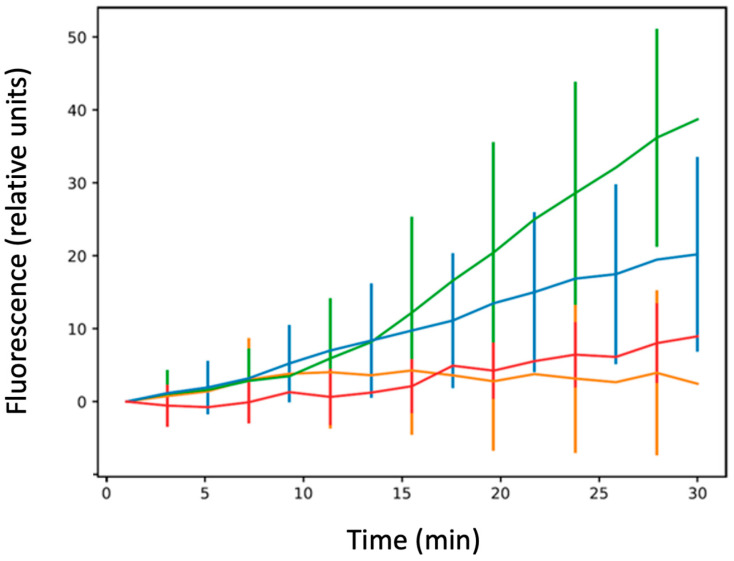
Ciprofloxacin accumulation at the single-cell level. Blue: AG100; green: AG100 ∆*ctr*; orange: AG100 ∆*hfq*; red: AG100/pBRpLacMicF. The results are shown as mean values from ~30 bacteria analysed for at least three different experiments (independent pre-cultures), with error bars indicating SD. The one-way ANOVA analysis of variance followed by a Tukey’s post hoc test was used to compare the means between strains AG100 vs. AG100∆*hfq*, AG100 vs. AG100∆*ctr*, AG100∆*hfq* vs. AG100∆*ctr*, and AG100 vs. AG100/pBRpLacMicF at the last recorded time point, which showed that they are all significantly different from one to the other with a *p*-value less than 0.01. See also Supplementary Table S1.

This effect was then confirmed using measurement of accumulation at the single-cell level (Figure 5). First, we noted that, due to the absence of a functional efflux pump and as expected, the level of CIP was higher in AG100A (devoid of AcrB), than in AG100 (bearing active AcrB, compare values in Figure 3 and Figure 5, respectively). This was expected as no CIP efflux occurs in the absence of the efflux pump, and thus the concentration of CIP was expected to be higher in AG100 ∆acrB than in AG100.

One main difference between single-cell and bulk measurements involves the AG100 ∆hfq strain. In the single-cell analysis, we observe that this strain poorly accumulates CIP. The CIP accumulation is the same for AG100 and AG100 ∆ctr for ~5 to 7 min, and then AG100 ∆hfq cells stopped accumulating the drug (Figure 5). One explanation could be due to bacterial death after the long exposure to the antibiotic. Note that hfq deletion may induce UV sensitivity in the strain [53] during image acquisition. This eventuality was tested by increasing the time delay between acquisition of the same bacteria (one set of images acquired at 5 min and a second one at 15 min). In this experiment, with a lower exposure to UV, the accumulation of AG100 ∆hfq strain was similar to the one shown on the orange curve of Figure 5 (Supplementary Figure S2).

**Figure 6:**
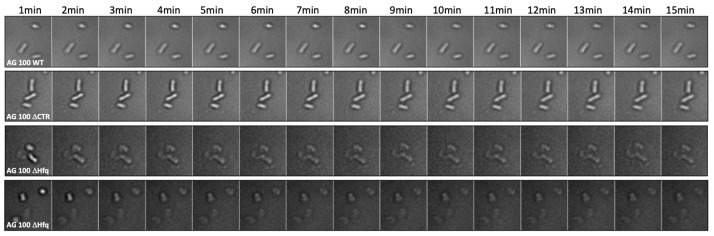
Morphological analysis of AG100 bacteria in the presence of CIP at the single-cell level. We clearly observe that AG100 ∆hfq bacteria lose their natural shape and become more spherical or strongly deformed. This effect is observed only for this specific strain and not for other AG100 (AG100 hfq+ and AG100 ∆ctr).

Using the imaging set up, we observed an unusual shape for AG100 ∆hfq cells, as compared with ∆ctr and WT strains (Figure 6). We also noted a very slow growth rate when cultured. In parallel, we evaluated bacterial survival using the LIVE/DEAD^TM^ BacLight^TM^ staining method. Survival was around 99% for AG100 hfq+ strain, while it was only ~85% for the AG100 ∆hfq strain, confirming a lower viability of this strain compared with others (Supplementary Figure S3). This lower viability was confirmed by plating both strains after 7 min of CIP exposure; more precisely, we observed 20.6% less colonies for ∆hfq strain, a value in agreement with LIVE/DEAD staining viability measurement (Supplementary Figure S3).

Next, as Hfq may modulate FQ import by an sRNA-dependent negative regulation of mRNA encoding outer membrane proteins (Omps) [54], we tested the effect of a lower abundance of porins on CIP accumulation. Among the porins regulated by sRNA and Hfq [38,39], the outer membrane OmpF protein is clearly involved in the influx of antibiotics, including FQ, and decreased amounts of OmpF caused resistance to multiple antibiotics, especially FQ [55,56]. To test the effect of MicF on CIP accumulation, we used a plasmid overproducing the MicF sRNA to reduce the expression of ompF by ~80% [46,57,58]. Under these conditions, accumulation of CIP was comparable to that of the WT strain in bulk analysis (Figure 4). In the single-cell analysis, the fluorescence increases slowly with time but remains significantly below the level observed in WT bacteria (Figure 5). The differences between results obtained in the bulk and single-cell experiments might arise from different time courses of the experiments, as the results appear comparable at short times. Furthermore, these differences may be too small to be seen in bulk given the variability of bacterial population, while this difference can be evidenced using the single-cell analysis. Note that we did not find a significant cell mortality or abnormal cellular shape in the case of impaired expression of the ompF gene, conversely to the AG100 ∆hfq strain (Figure 6).

## 4. Discussion

Previous studies reported that Gram-negative bacteria, *E. coli* or *Aeromonas veronii*, devoid of Hfq are more sensitive to antibiotics [36,37]. Furthermore, deleting the *hfq* gene results in an increase in antibiotic accumulation [37]. The same study evidenced that deletion of the *acrB* gene, a component of the RND efflux pump, attenuates the effect of the *hfq* deletion on bacterial antibiotic sensitivity [37]. The level of the AcrB protein in the ∆*hfq E. coli* strain was greatly reduced compared with the WT strain, without affecting the activity of the *acrAB* operon promoter, indicating that Hfq regulates the expression of *acrB* at the post-transcriptional level [37]. The results of this study are thus compatible with the previous analysis, indicating an increase in the accumulation of antibiotics in the absence of Hfq [37]. Furthermore, here we quantify this effect, an aspect that was missing in Yamada et al., 2010 [37]. Our results indicate that Hfq has little or no influence on the accumulation of ciprofloxacin depending on the presence or absence of AcrB. This was quantified by calculating the CIP accumulation ratio of AG100 *hfq*+/AG100A *hfq*+ and that of AG100 ∆*hfq*/AG100A ∆*hfq*, and both are around 1.3. The difference in accumulation between AG100 and AG100 ∆*hfq* was explained by the fact that Hfq negatively regulates the expression of *acrB*, hence, the difference in accumulation between AG100 and AG100∆*hfq*. Here we show that there is still an effect of the deletion of *hfq* in AG100A; one explanation might be that Hfq influences the expression of proteins other than AcrB.

We rule out the possibility that Hfq may regulate other RND pumps, as previous results have repeatedly shown that the accumulation of FQs was identical in AG100A and in AG100 grown in the presence of Carbonyl Cyanide m-ChloroPhenyl hydrazone (CCCP) (M. Masi, personal communication). CCCP proton pumps are known to inactivate all RND pumps by dissipating the transmembrane potential needed by the efflux pumps [41]. Another possibility would be the intervention of an ABC transporter such as MacAB-TolC, even if such a transporter has a poor affinity for FQs [59].

We thus propose that the main effect of Hfq on FQ accumulation is due to its role in the regulation of the outer membrane porin OmpF production. This porin has previously been identified as a key player in FQ antibiotics influx, and impaired expression of ompF indeed induced resistance to FQ [55,56].

An intriguing result was that in the presence of CIP, altered shapes of cells were observed in the mutant devoid of the Hfq protein only in the presence of the AcrAB pump, not in its absence. Indeed, we observed that these ∆*hfq* cells lose their natural shape to become more spherical or strongly deformed in the presence of CIP. The mechanism of this phenomenon remains to be elucidated. However, significant changes in bacterial cell morphology might be related to either disruption of the integrity of the cell envelope or dysregulation of DNA replication [60,61,62]. Moreover, Hfq is a direct or indirect regulator of expression of many genes, including morphogenes (*mreB*, *bolA*) [62]. Therefore, it is likely that in the absence of Hfq, cells might have difficulty in maintaining their shape as a result of the dysregulation of the networks of gene-expression events. On the other hand, as the effects on the cell shape in Hfq-deficient cells subjected to CIP action were evident only in the presence of AcrAB, it is likely that AcrAB is involved in the specific Hfq-dependent mechanism(s). One might speculate that Hfq could positively regulate expression of *acrB*, thus, in the mutant with deletion of this gene, the effects of the absence of Hfq on the cell shape are negligible, as the AcrAB pump would not be produced efficiently anyway. If it remains to be elucidated why CIP is able to cause changes in the ∆*hfq* cell shape only in the presence of the pump, it is likely that there are interactions between ArcAB and CIP, which in the presence of Hfq leads to an effective efflux of the antibiotic, but in the absence of Hfq become toxic to cells. If this is true, it would be tempting to speculate that Hfq might control the expression of gene(s) whose product(s) are involved in the effective transportation of CIP outside the cell through the AcrAB system.

**Figure 7:**
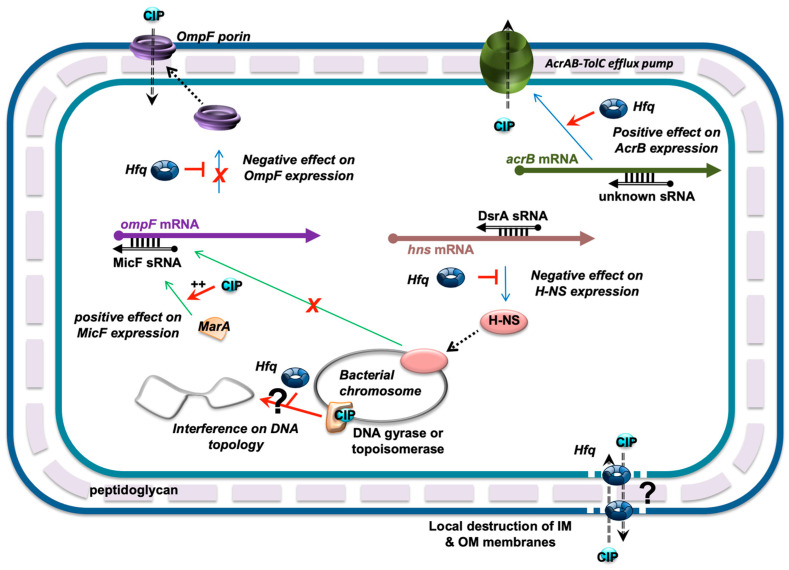
Network of Hfq-dependent regulations influencing FQ fluxes. MicF was previously shown to be regulated at the transcriptional level by MarA and H-NS transcription factors [65,66]. Since *hns* translation is repressed by DsrA and Hfq (note that the nature of DsrA:*hns* mRNA complex is still unclear and that it has been proposed that the 3′ UTR of *hns* mRNA could be required for its post-transcriptional regulation [67]), Hfq may also influence *micF* transcription negatively. Additionally, *ompF* mRNA translation is also directly repressed by MicF sRNA with the help of Hfq. In parallel, Hfq positively regulates the expression of *acrAB* (coding for components of the efflux pump) at the post-transcriptional level [37]. Finally, Hfq could also directly influence CIP fluxes independently of its role in the sRNA-based regulation, as it creates pores in IM and OM membranes [68,69]. Finally, interference between Hfq and DNA-gyrase or topoisomerase of DNA topology may also occur and influence DNA-related processes such as transcription. CIP is depicted as a blue circle. sRNA regulators controlling mRNAs are shown as black open arrows; Hfq is represented by a toroidal hexamer; mRNAs are depicted as thick black lines; 5′ and 3′ ends of the mRNA are depicted by a “ball and arrow head”, respectively; H-NS and MarA are shown as orange and pink ellipses, respectively; transcriptional regulations are represented by thin green lines; translational regulations are shown as thin blue lines; positive and negative regulations are indicated by red arrows and horizontal bars, respectively; dotted line symbolizes peptidoglycan (PG) between outer (OM) and inner (IM) membranes.

Concerning OmpF regulation, the expression of *ompF* is regulated at both the transcriptional and post-transcriptional levels. For the latter, the translation of *ompF* is inhibited by an antisense sRNA called MicF. The 5′ end of MicF RNA (~25 nucleotides) anneals to the *ompF* 5′ untranslated region of mRNA and forms a duplex (Figure 7). In this duplex, MicF sRNA sequesters the ribosome-binding site (*rbs*, Shine-Dalgarno sequence) and the AUG start codon of the *ompF* mRNA, thereby inhibiting its translation [63]. This repression fully depends on Hfq, as the MicF:*ompF* duplex is imperfect and proper annealing requires the protein cofactor [64].

Under normal conditions of growth, the production of MicF sRNA occurs at a low level, but the presence of antibiotics increases transcription of *micF* [56]. The expression of *micF* is indeed increased by the MarA transcriptional activator in the presence of CIP, resulting in impairment of the *ompF* expression [65,66]. Reduced expression of *ompF* thus favors the resistance to CIP. Conversely, strains devoid of Hfq are more sensitive to FQ as no negative regulation of *ompF* by MicF occurs [37].

Note that other factors controlled by Hfq may also be necessary for the regulation of *ompF* expression. For instance, a transcriptional regulator H-NS regulates *micF* transcription [70] and Hfq negatively regulates the expression of *hns* at the post-transcriptional level through DsrA sRNA [71]. Thus, OmpF levels are decreased in an *hns* mutant, but increased in an *hfq* mutant, as H-NS translation is no longer repressed by Hfq [72].

Additionally, we also reported that the presence of a truncated form of Hfq devoid of its C-terminus region results in a more effective accumulation of CIP (in both *acrB*+ and *acrB-* backgrounds). The effect of the *ctr* deletion on CIP accumulation is intermediate between that in the *hfq*+ and ∆*hfq* strains. Previous studies indicated that Hfq-CTR may be dispensable for riboregulation [25]. However, here we confirm the results of other studies suggesting that the CTR region of Hfq may play a role in sRNA-based regulation [26,27]. Furthermore, this region of the protein is amyloid-like [24] and Hfq, due to its CTR region, may affect membrane integrity (both IM and OM) [68,69]. Therefore, one can imagine that the Hfq protein could also directly influence CIP fluxes independently of its role in sRNA-based regulation (Figure 7). Additionally, crosstalk between Hfq effects on DNA metabolism [28], and that of FQ that acts on DNA-gyrase and inhibits DNA supercoiling relaxation, may also occur (Figure 7).

## 5. Conclusions

The main result of the current study is that Hfq has little or no influence on the accumulation of ciprofloxacin depending on the presence or absence of the AcrB-containing efflux pump. We indeed confirm results of previous reports [37], but show additionally that there is an effect of the deletion of *hfq* in AG100A (devoid of AcrB), Hfq also significantly influences the expression of proteins other than AcrB involved in quinolone fluxes. We present evidence using a strain expressing MicF sRNA, that the OmpF porin could be one of these proteins. Nevertheless, no final conclusions about the role of OmpF (and MicF) can be drawn as the MicF-based regulation imposes the presence of Hfq, and the analysis of the OmpF effect in the AG100 ∆*hfq* strain would also be needed to draw this conclusion. In addition, Hfq may influence the expression of other outer-membrane porins [38,39]. We thus plan to analyse a series of mutants deleted of various *omp* genes, including those encoding OmpF but also OmpC or OmpD [73], to exert a full repression of the Omps in the absence of Hfq that may reveal new mechanisms of regulation of FQ fluxes by sRNA and Hfq.

## Figures and Tables

**Table 1:** *E. coli* strains used in this study.

Strain	Description	Efflux Pump	Hfq	Source
AG100	AG100	+	+	[43]
AG100A	AG100 a*crB*::*kan*	−	+	[44]
AG100 ∆*hfq*	AG100 *hfq*::*cm*	+	−	This study
AG100 ∆*ctr*	AG100 *hfq72-cm*	+	±	This study
AG100A ∆*hfq*	AG100 a*crB*::*kan hfq*::*cm*	−	−	This study
AG100A ∆*ctr*	AG100 a*crB*::*kan hfq72-cm*	−	±	This study
AG100 + p*MicF*	AG100/pBRpLac*MicF*	+	+	This study

## Data Availability

The data that support the findings of this study are available on request from the corresponding authors.

## Supplementary Materials

The following supporting information can be downloaded at: https://www.mdpi.com/article/10.3390/microorganisms12010053/s1, Supplementary Figure S1: Normal distribution of bacterial populations based on single-cell analysis; Supplementary Figure S2: CIP accumulation at the single-cell level with lower exposure to UV; Supplementary Figure S3: Evaluation of AG100A ∆*hfq* strain survival using a Live/Dead test and plating after CIP exposition; Supplementary Table S1: Ciprofloxacin accumulation values at the single-cell level in bacteria.
