# Supplementary material for: Role of the Bacterial Amyloid-like Hfq in Fluoroquinolone Fluxes
Source: Microorganisms. 2023 Dec 28;12(1):53. doi: 10.3390/microorganisms12010053 (PMC10819720; doi:10.3390/microorganisms12010053)
Supplement: Supplementary file 1 [file microorganisms-12-00053-s001.zip › microorganisms-2772825-supplementary.pdf]

Sup Fig. S1: Normal distribution of bacterial populations based on single cell analysis

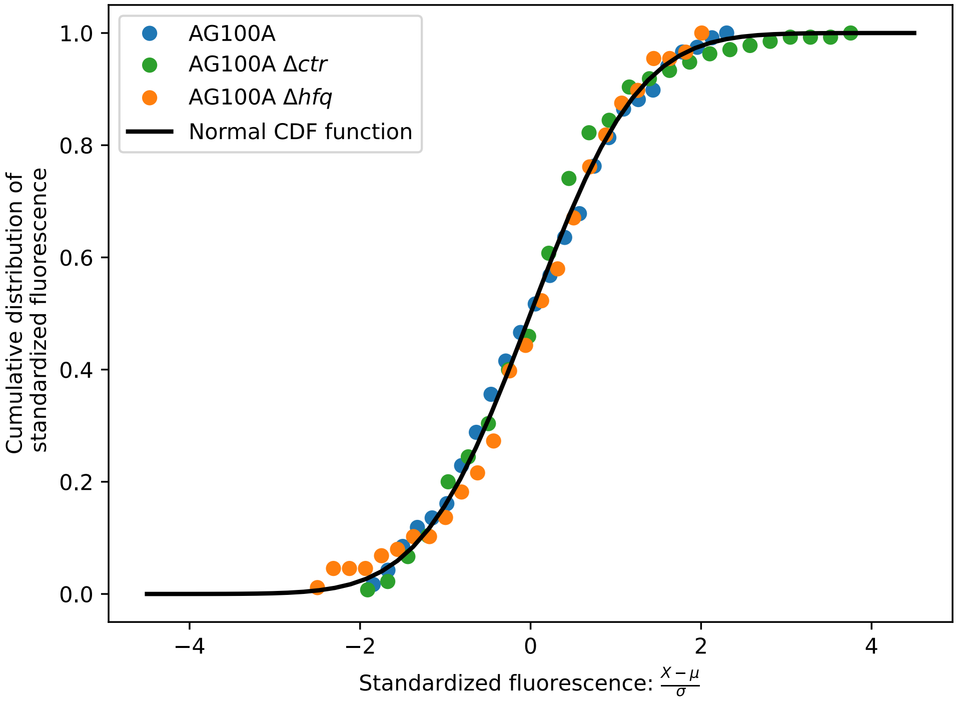

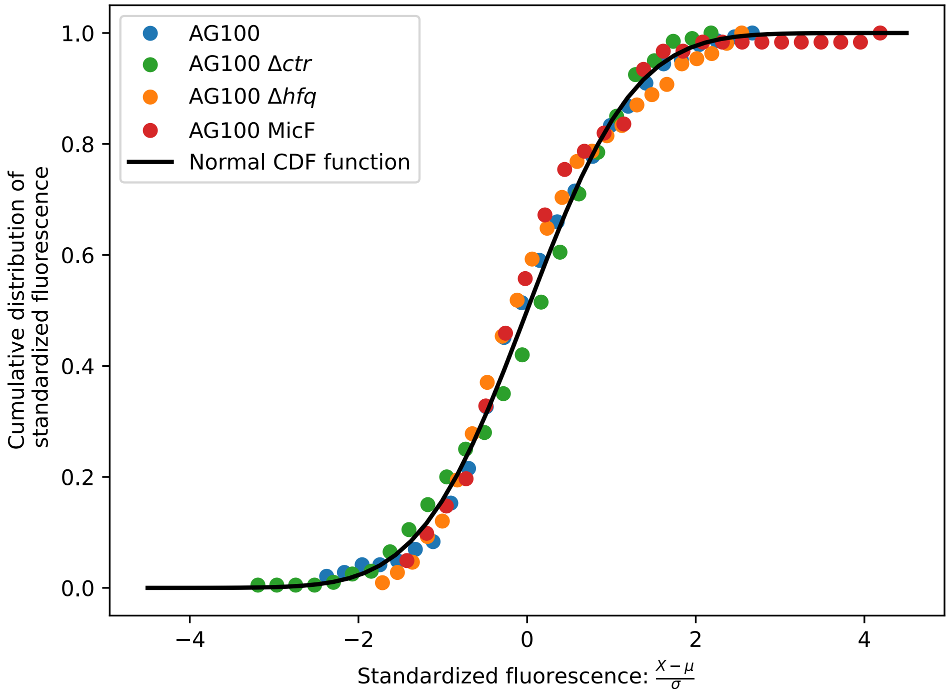

Sup Fig. S2: CIP accumulation at the single cell level with lower exposure to UV

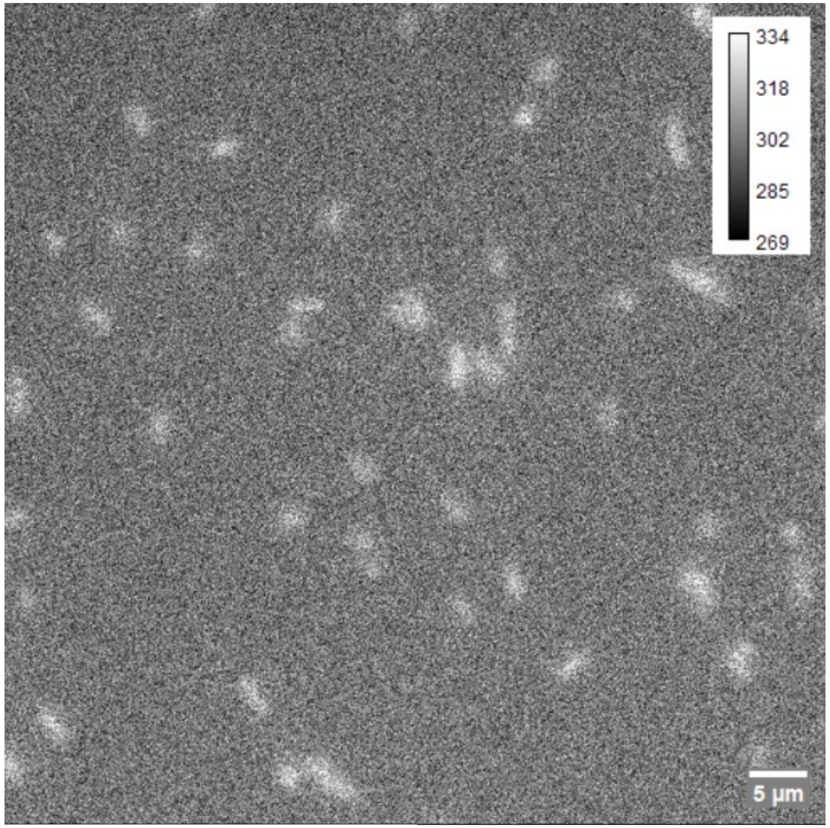

AG100 ΔHfq 5min	
**mean flurorescence**	**std**
315	10,61

Mean fluorescence : 315
Standard deviation : 10,61

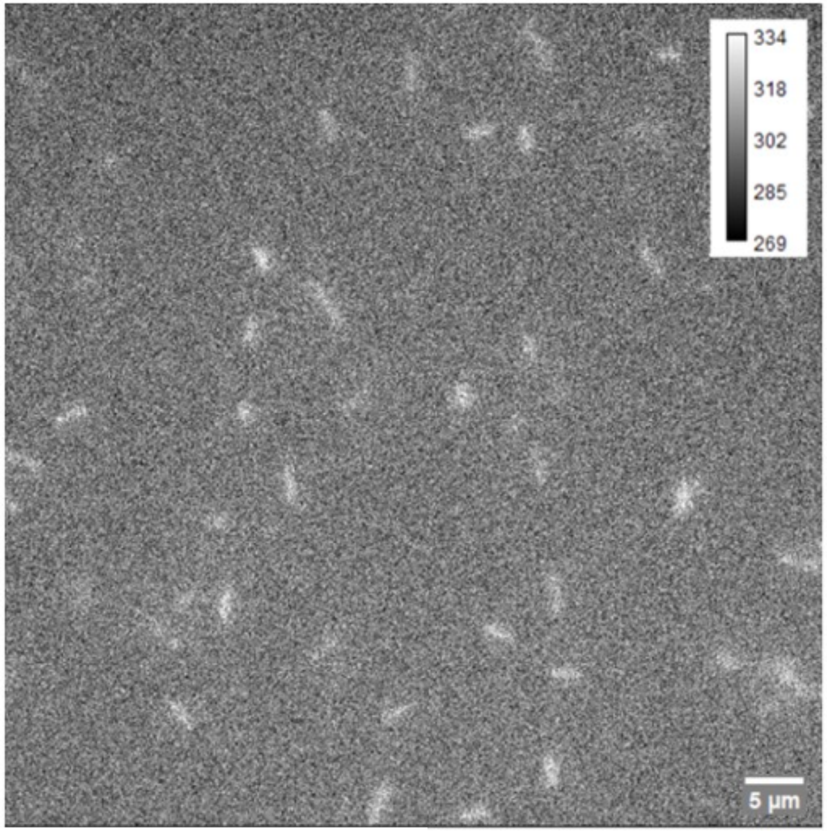

AG100 ΔHfq 15min	
**mean fluorescence**	**std**
312,1	13,64

Mean fluorescence : 312,1
Standard deviation : 13,64

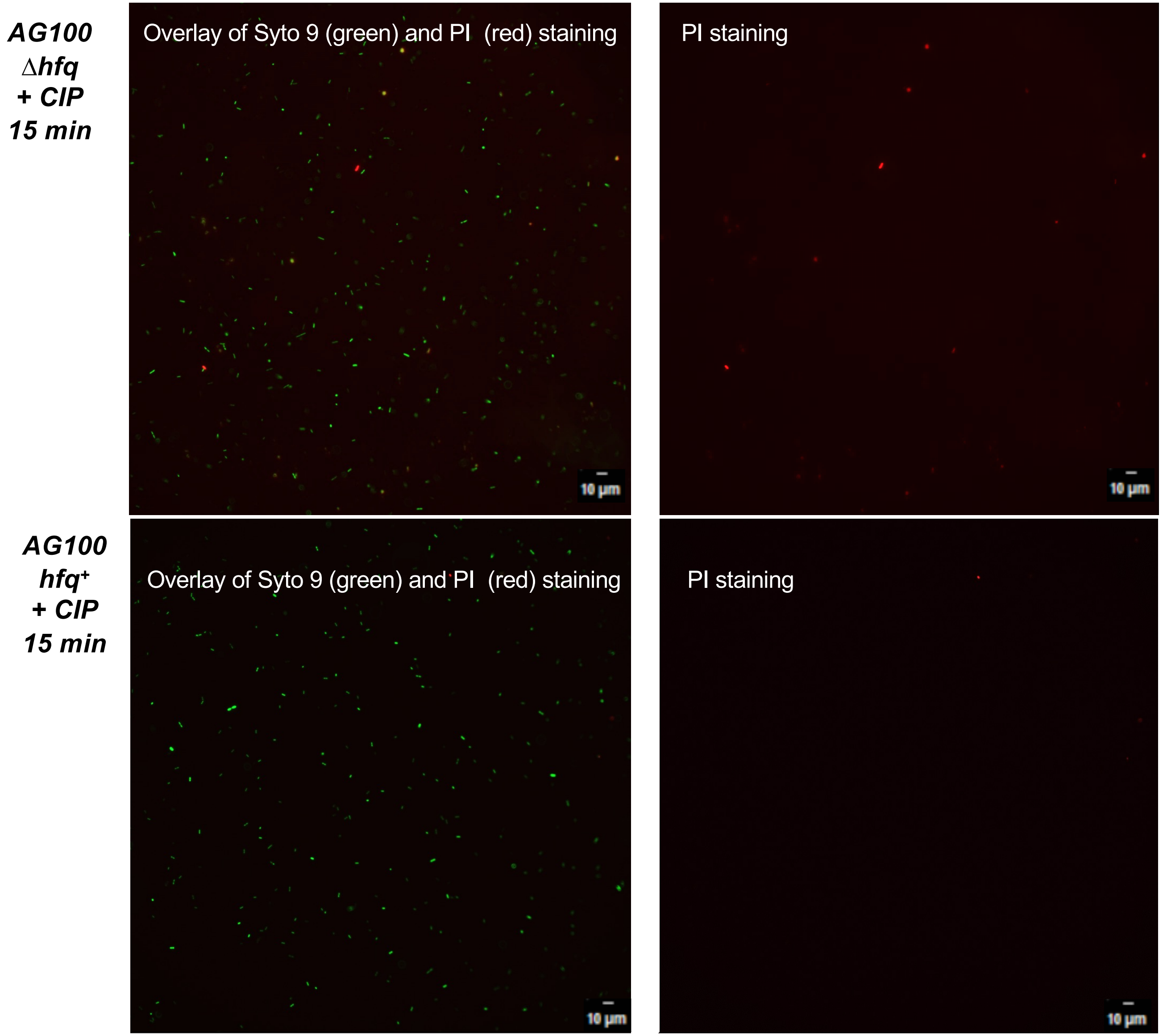

Sup Fig S3: *Left:* Evaluation of AG100 Δ*hfq* strain survival using Live/Dead staining. *Right:* Plating of AG100 Δhfq and WT strain after 7 min of incubation with CIP (only one example of the triplicates is shown). Note that AG100 Δ*hfq* colonies are smaller than those of AG100.

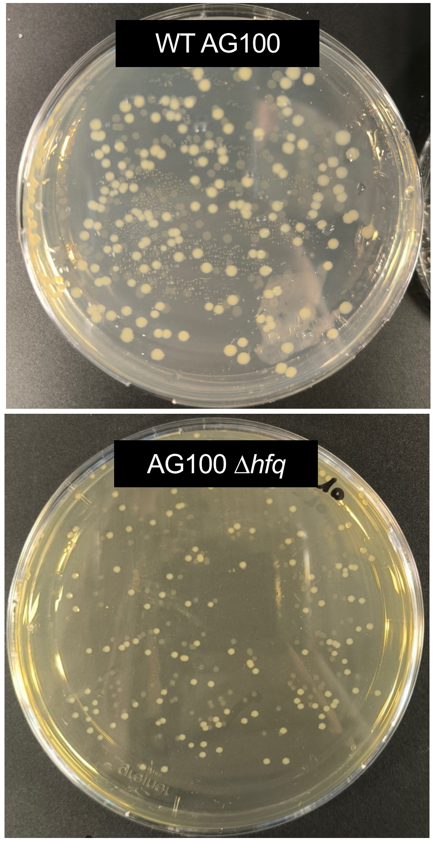

Sup table S1: Ciprofloxacin accumulation values at the single cell level in bacteria
(Std = standard deviation)

	AG100 WT	140 bacteria	AG100 ΔCTR	255 bacteria	AG100 ΔHfq	104 bacteria	AG100/pBRpLacMicF	54 bacteria	AG100A WT	114 bacteria	AG100A ΔCTR	131 bacteria	AG100A ΔHfq	84 bacteria
**Time (min)**	**mean**	**std**	**mean**	**std**	**mean**	**std**	**mean**	**std**	**mean**	**std**	**mean**	**std**	**mean**	**std**
**3**	1,161	2,988	1,027	3,301	0,754	3,310	-0,548	2,859	-9,909	11,192	4,423	35,482	-18,626	35,074
**5**	1,949	3,637	1,594	3,991	1,409	4,135	-0,763	2,844	1,218	12,936	25,807	43,015	4,547	43,402
**7**	3,193	4,464	2,846	4,452	3,006	5,650	-0,088	2,902	14,594	16,574	48,231	44,866	33,900	48,209
**9**	5,242	5,273	3,496	6,046	3,820	6,678	1,297	3,007	26,088	19,657	70,631	47,235	66,250	52,926
**11**	7,009	6,286	5,930	8,222	4,034	7,662	0,641	3,856	37,733	22,870	93,309	51,604	106,319	66,011
**13**	8,369	7,812	8,168	10,269	3,617	7,753	1,231	3,154	48,555	26,392	110,477	55,759	151,431	80,179
**15**	9,752	8,643	12,216	13,146	4,271	8,828	2,115	3,638	55,767	27,848	127,028	57,196	184,511	85,157
**17**	11,117	9,195	16,583	14,607	3,600	9,054	4,926	3,962	63,241	30,031	143,207	58,804	208,047	90,245
**19**	13,485	10,128	20,451	15,142	2,789	9,510	4,243	3,834	68,465	30,731	159,668	62,594	230,670	92,388
**21**	15,011	10,949	24,995	15,310	3,772	10,228	5,545	4,634	73,094	33,076	172,493	65,076	244,343	96,351
**23**	16,861	12,089	28,581	15,256	3,163	10,220	6,441	4,453	77,750	34,519	184,490	68,124	255,428	100,044
**25**	17,481	12,306	32,106	15,583	2,659	10,916	6,129	5,097	80,365	35,315	192,716	71,212	261,737	100,563
**27**	19,470	12,760	36,191	14,926	3,943	11,302	8,014	5,432	81,507	35,291	195,708	74,158	290,037	103,548
**30**	20,203	13,348	38,716	15,435	2,445	11,485	8,952	4,186	83,071	36,782	199,767	76,520	308,009	105,757
